# Altered amygdala resting-state functional connectivity in anxiety disorders: a coordinate-based meta-analysis

**DOI:** 10.1017/S0033291726104310

**Published:** 2026-05-21

**Authors:** Lu Lu, Haoran Xu, Rui Wang, Baoshuai Zhang, Baolin Wu, Lisha Zhang, Xiao Li, Qiyong Gong, Jeffrey R. Strawn

**Affiliations:** 1 Department of Radiology, Huaxi MR Research Center (HMRRC), Institute of Radiology and Medical Imaging, Psychoradiology Key Laboratory of Sichuan Province, West China Hospital of Sichuan University, Chengdu, Sichuan, China; 2 Research Unit of Psychoradiology, Chinese Academy of Medical Sciences, Chengdu, Sichuan, China; 3 Department of Interventional Therapy, National Cancer Center/National Clinical Research Center for Cancer/Cancer Hospital, Chinese Academy of Medical Sciences and Peking Union Medical College, Beijing, 100021, China; 4 Department of MRI, The First People’s Hospital of Yunnan Province, Kunming, Yunnan, China; 5 Xiamen Key Lab of Psychoradiology and Neuromodulation, Department of Radiology, West China Xiamen Hospital of Sichuan University, Xiamen, Fujian, China; 6 Department of Psychiatry and Behavioral Neuroscience, College of Medicine, University of Cincinnati, Cincinnati, Ohio, USA

**Keywords:** ACC, amygdala, anxiety disorder, meta-analysis, resting-state functional connectivity

## Abstract

**Background:**

Anxiety disorders are associated with disrupted amygdala connectivity; however, resting-state functional MRI studies have reported heterogeneous findings. To clarify these inconsistencies, we conducted a meta-analysis of amygdala-based connectivity studies.

**Methods:**

A systematic search of Embase, PubMed, and Web of Science was performed through December 26, 2025. Studies comparing amygdala-based whole-brain resting-state functional connectivity in patients with anxiety disorders versus healthy controls were included. Meta-analysis was conducted with the latest software – Seed-based d Mapping with Permutation of Subject Images (SDM-PSI), which employs voxel-wise tests and multiple corrections to minimize false positives. Subgroup analyses were performed to examine differences by age and hemisphere.

**Results:**

Fifteen datasets (378 patients, 405 controls) were included. Compared to healthy controls, patients with anxiety disorders had decreased amygdala-anterior cingulate cortex (ACC, *g* = −0.54, 95% confidence interval [CI]: −0.73 to −0.35) connectivity and increased connectivity with the left superior temporal gyrus (*g* = 0.46, 95% CI: 0.27–0.65), middle temporal gyrus (*g* = 0.38, 95% CI: 0.19–0.57), and cuneus (*g* = 0.35, 95% CI: 0.17–0.53). After threshold-free cluster enhancement correction, only reduced amygdala-ACC connectivity remained significant (*g* = −0.54, 95% CI: −0.73 to −0.35). Subgroup analyses confirmed this effect was driven mainly by adult patients and the left amygdala.

**Conclusions:**

Reduced connectivity between the left amygdala and the ipsilateral ACC was the most robust neuroimaging marker of anxiety disorders, which suggests a lateralized vulnerability. By applying updated analytic methods, this study refines our understanding of the neuropathology of anxiety disorders and provides a potential primary target for biomarker development and novel interventions.

## Introduction

Anxiety disorders are among the most prevalent psychiatric illnesses, currently affecting ~7.3% of the global population and with a lifetime prevalence of nearly 31% in the United States (Merikangas et al., 2010). Clinically, anxiety disorders are characterized by persistent, excessive, and uncontrollable anxiety and fear, along with exaggerated responses and avoidance behaviors related to anxiety-provoking situations (Association, 2013). These disorders typically emerge in early adulthood or adolescence (Penninx, Pine, Holmes, & Reif, 2021) and generally persist into adulthood. Due to their high incidence, comorbidity, and chronicity, anxiety disorders are the ninth leading cause of global disability and inflict substantial economic and health-related burden (GBD 2016 Disease and Injury Incidence and Prevalence Collaborators, 2017), and globally contribute to significant economic and health burdens. Despite their significant impact, the pathophysiology of anxiety disorders is poorly understood. Altered amygdala connectivity is directly linked to hallmark symptoms of anxiety disorders – such as hypervigilance, exaggerated threat appraisal, and impaired regulation of fear – which in turn contribute to avoidance behaviors, functional impairment, and chronic course.

Disrupted functional connectivity patterns have been described in anxiety disorders, particularly the amygdala, the hub of the emotion response and regulation circuits (Ding et al., 2011; Etkin et al., 2009; Madonna, Delvecchio, Soares, & Brambilla, 2019; Sergerie, Chochol, & Armony, 2008; Sylvester et al., 2012). Abnormal resting-state functional connectivity (rsFC) between the amygdala and multiple brain regions has been reported, including the dorsolateral prefrontal cortex (Jung et al., 2018; Liu et al., 2015), anterior cingulate cortex (ACC) (Du et al., 2021; Makovac et al., 2016; Prater et al., 2013; Roy et al., 2013; Wang et al., 2021), insula (Du et al., 2021; Jung et al., 2018; Li et al., 2016; Liu et al., 2015; Roy et al., 2013; Wang et al., 2021), middle temporal gyrus (MTG) (Li et al., 2016; Pannekoek et al., 2013), superior temporal gyrus (STG) (Du et al., 2021; Liu et al., 2015; Roy et al., 2013; Wang et al., 2021), and precuneus (Ergül et al., 2019; Jung et al., 2018; Toazza et al., 2016). However, findings across studies are inconsistent. For example, in adults with generalized anxiety disorder (GAD), one study reported reduced rsFC between the amygdala and inferior frontal gyrus compared to healthy controls (Du et al., 2021), whereas another study reported increased rsFC between the amygdala and inferior frontal gyrus (Li et al., 2016). Similarly, in adolescents with GAD, increased rsFC was observed between the right amygdala and cerebellum (Liu et al., 2015), while in another study, decreased connectivity was observed in the same circuit (Roy et al., 2013). Further, in adolescents with GAD, one study observed increased rsFC between the amygdala and medial prefrontal cortex (Roy et al., 2013), while another study of drug-naïve GAD patients showed decreased connectivity between the basolateral amygdala and medial prefrontal cortex (Wang et al., 2021). These discrepancies may be attributable to differences in sample size, demographics, illness duration, symptom severity, medication status, and analytic strategies (e.g. correction methods), which present major barriers to identifying consistent neurobiological patterns and translating neuroimaging findings into clinical practice (Baumel et al., 2022). In this context, it is essential to synthesize findings of the amygdala connectivity from existing studies to help us better understand the mechanism of anxiety disorder.

A systematic review of amygdala-based functional connectivity (FC) in SAD, incorporating both resting-state and task-based fMRI studies, reported increased amygdala connectivity with prefrontal and orbitofrontal regions, reduced connectivity with parietal regions, and inconsistent findings in the temporal cortex (Brühl, Delsignore, Komossa, & Weidt, 2014). Another systematic review in GAD reported increased rsFC between the amygdala and widespread regions, including the prefrontal cortex, insula, superior temporal gyrus, and precentral gyrus (Kolesar, Bilevicius, Wilson, & Kornelsen, 2019). However, these reviews combined data across different imaging modalities and lacked quantitative analysis, which limited the interpretability and generalizability of their findings. Meta-analysis is an effective method to quantitatively integrate findings from small-sample neuroimaging studies and identify robust alteration patterns of disorders. It also enables us to analyze the associations between neuroimaging results and clinical characteristics (Radua & Mataix-Cols, 2009). To date, only two meta-analyses have examined amygdala-based FC alterations in anxiety disorders, both using the activation likelihood estimation (ALE) approach. One included 10 studies and reported hypoconnectivity between the right amygdala and dorsomedial prefrontal cortex, and between the left amygdala and ventromedial prefrontal cortex (Xu et al., 2019). However, this meta-analysis included studies that examined the relationship between anxiety and amygdala FC in healthy participants, and studies examined amygdala FC with specific regions instead of the whole brain (Xu et al., 2019). The second meta-analysis included 14 studies (10 studies reported healthy > anxious and 10 studies reported anxious > healthy findings), and found hypo-connectivity between amygdala and medial prefrontal cortex and ACC (Zugman et al., 2023). This meta-analysis also included the original studies without direct whole-brain, two-group comparisons, and included panic disorder whose clinical characteristics, risk factors, and phenomenology differ from GAD and SAD (Zugman et al., 2023). Importantly, both meta-analyses were vulnerable to selective bias inherent to the ALE approach, which includes only studies reporting significant findings (i.e. anxious > healthy or healthy > anxious), thereby excluding studies with null findings.

Studies in healthy individuals indicate that the left and right amygdala connectivity are asymmetric, with each differently contributing to psychopathology, including anxiety disorders. For example, prior reviews indicate greater left amygdala activation in emotional processing compared to the right amygdala (Baas, Aleman, & Kahn, 2004). In major depressive disorder, one study found that left amygdala hypoconnectivity involved more brain regions than the right amygdala (Muetzel et al., 2016). Similarly, in SAD (Jung et al., 2018), the left amygdala showed increased connectivity with regions such as the anterior insula and fusiform gyrus, while the right amygdala exhibited decreased connectivity with the medial frontal cortex. This lateralization also appears to extend to treatment response in anxiety disorders. For example, in our previous double-blind, placebo-controlled trial of adolescents with anxiety disorders, escitalopram increased the left amygdala connectivity but not the right amygdala during resting state, and decreased right amygdala-ACC connectivity but increased left amygdala connectivity with the angular gyrus during the emotion-processing task (Lu et al., 2021, 2022). Taken together, these findings suggest that the right and left amygdala connectivity changes in anxiety disorders are different and should be examined separately when performing meta-analyses.

In this study, we aimed to systematically synthesize and integrate previous research on amygdala FC abnormalities in anxiety disorders using the latest version of Seed-based d Mapping with Permutation of Subject Images (SDM-PSI) (Albajes-Eizagirre, Solanes, Vieta, & Radua, 2019). This approach builds on earlier coordinate-based meta-analytic techniques (e.g. activation likelihood estimation, multilevel kernel density analysis [MKDA]) to overcome the selective bias of ALE approaches, by allowing inclusion of both significant and nonsignificant findings, which reduces bias and improves accuracy and sensitivity (Radua et al., 2012). Earlier versions of SDM relied on voxel-wise or cluster-wise statistics that were often overly conservative or excessively liberal and were unable to correct for family-wise error (FWE) using true permutation testing. The updated SDM-PSI overcomes these limitations by implementing permutation of subject images based on the Freedman–Lane approach, along with Threshold-Free Cluster Enhancement (TFCE). Additionally, SDM-PSI incorporates Meta NSUE-based multiple imputation to reduce bias in effect size estimation derived from peak coordinates and applies Rubin’s rules to appropriately combine imputed datasets. These enhancements improve sensitivity and specificity while reducing false positives from inconsistent thresholding. Applied to the same dataset, SDM-PSI consistently produces more conservative, robust results than earlier versions, particularly for findings that remain significant after TFCE correction.

With these considerations in mind, we used SDM-PSI to examine amygdala-based whole-brain connectivity abnormalities in anxiety disorders. Our goal was to identify amygdala connectivity changes in anxiety disorders and refine our understanding of these disorders. This is particularly relevant for fear-based anxiety disorders – generalized, social, and separation anxiety disorders – which share core features of hypervigilance, excessive worry, and heightened threat responsivity. These disorders also exhibit overlapping risk factors, comorbidity patterns, and similar pharmacologic and psychotherapeutic treatment responses, making them well-suited for pooled neuroimaging analyses (Salum et al., 2011; Verduin & Kendall, 2003). We therefore conducted a quantitative meta-analysis across these disorders. Recognizing potential lateralization effects, we then performed subgroup analyses using the left and right amygdala as seeds separately to explore differential connectivity patterns. We also compared amygdala functional connectivity between adults and adolescents to investigate developmental trajectories of anxiety disorders. Based on prior work, we hypothesized that individuals with anxiety disorders would show aberrant amygdala rsFC with key brain regions, such as the prefrontal cortex, ACC, and temporal cortex. We further hypothesized that these alterations would be lateralized.

## Methods

### Search strategy

A comprehensive literature search was carried out using the Web of Science, PubMed, and Embase databases on December 26, 2025 (including studies in press), focusing on seed-based FC studies of anxiety that used the amygdala as the seed. Keyword searches were conducted using the “(anxiety OR anxious) AND (rest*) AND (connect*) AND (amygdala*)” (Supplementary Material 1). These searches yielded 2,595 original or review articles. After removing the duplicates (*n* = 1,091), 1,504 studies were reviewed, with 101 evaluated in full. In addition, the references of the retrieved studies and related review articles were also manually searched.

### Study eligibility criteria

Eligible studies were required to include both a healthy control group and a group of patients with anxiety disorders diagnosed according to the *Diagnostic and Statistical Manual of Mental Disorders* (*DSM*), as reported in the original studies. No restriction was imposed on a specific DSM version during study selection. We focused on social anxiety disorder, generalized anxiety disorder, and separation anxiety disorder, as these conditions share substantial clinical overlap and are frequently comorbid. Only studies that employed resting-state functional MRI and used the amygdala as a seed region – whether defined by a sphere or a mask of subregional or whole amygdala – were considered. Furthermore, studies had to conduct functional connectivity analyses between the amygdala and whole-brain voxels, and results were required to be provided in Montreal Neurological Institute (MNI) or Talairach coordinates.

Studies were excluded if they lacked a control group or an anxiety disorder group or if they examined posttraumatic stress disorder, obsessive-compulsive disorder, panic disorder, specific phobia, or subthreshold anxiety that did not meet the DSM diagnostic criteria for anxiety. We also excluded studies that did not use whole-brain resting-state functional connectivity methods, such as those relying on independent component analysis, graph theory, or effective connectivity approaches. Additional exclusion criteria included non-original publications (e.g. conference abstracts, reviews, and meta-analyses), studies with overlapping samples (in which case only the study with the largest sample size was retained), and studies for which between-group coordinates could not be retrieved despite attempts to contact the authors. Only peer-reviewed publications were included.

**Figure 1.**
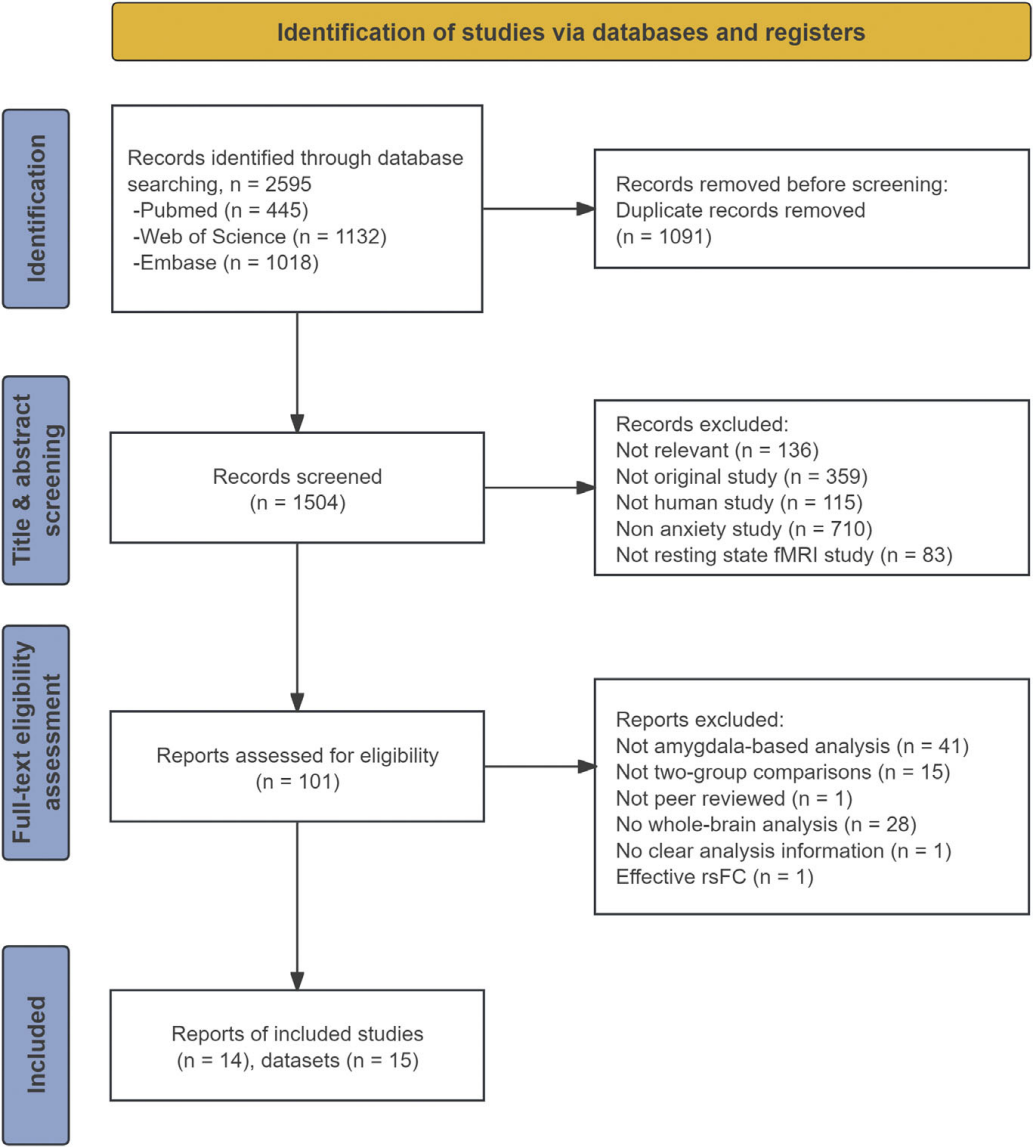
Preferred Reporting Items in Systematic Reviews and Meta-Analyses (PRISMA) flow diagram of literature search and study selection.

The study selection process followed Preferred Reporting Items for Systematic Reviews and Meta-Analyses (PRISMA) guidelines (Moher et al., 2009), and the detailed procedures for study selection are shown in Figure 1. The study was registered in the Prospective Register of Systematic Reviews (PROSPERO) under the number CRD420251019885.

### Data extraction

After identifying the original articles that met the inclusion criteria, we extracted relevant data from all original studies, including sample sizes, clinical diagnoses, age, gender, comorbidities, peak coordinates of inter-group comparisons, and effect sizes, for the subsequent meta-analysis. The literature search, title and abstract screening, full-text eligibility assessment, and data extraction for this study were conducted independently by two researchers and cross-verified (Xu and Lu). Discrepancies arising during title/abstract screening, full-text eligibility assessment, or data extraction were resolved through discussion between Xu and Lu. Any disagreements that remained unresolved were adjudicated by a third author (Wang) until consensus was achieved. All included studies were also assessed for methodological quality and potential sources of bias using a 12-point Imaging Methodology Quality Assessment Checklist (Shepherd et al., 2012). The checklist and individual study scores are provided in Supplementary Material 2 and Table 1.

**Table 1.** Demographic and clinical characteristics of the studies included in the meta-analysis

	Study	AD subjects						HC				Analytic methodology					
		*N*	Age	Females *N* (%)	Primary diagnosis	Anxiety severity	Diagnostic criteria	*N*	Age	Females *N* (%)	Anxiety severity	Software	Scanner	FWHM (mm)	Threshold	Seed definition method	Quality score
1	Hamm et al., 2014	33	13.9 (3.1)	67	GAD and SAD	PARS:22	DSM-IV	23	14.6 (3.9)	57	PARS:2	SPM8	3 T	8	FWE	AAL atlas	11
2	Li et al., 2016	21	39.9 (12.2)	33.3	GAD	HAMA:18.6	DSM-IV	22	38.1 (10.3)	36.4	HAMA:0.76	SPM8	3 T	9.5	AlphaSim	6 mm sphere	10.5
3	Liu et al., 2015	26	15.5 (1.5)	61.5	GAD	SCARED:37.12	DSM-IV	20	15.6 (1.7)	55.0	SCARED:16.55	DPARSFA2.0	3 T	NA	AlphaSim	6 mm sphere	10
4	Makovac.et al., 2016	19	29.5 (6.9)	89.5	GAD	PSWQ:54.73	DSM-IV	21	28.7 (9.5)	85.7	PSWQ:41.81	SPM8	1.5 T	NA	FWE	NA	10
5	Pannekoek et al., 2013	12	34.8 (8.8)	58.3	SAD	FQ:20.8	DSM-IV	12	34.0 (7.2)	58.3	FQ:4.5	FSL	3 T	6	FWE	4 mm sphere	10.5
6	Prater et al., 2013	20	25.9 (5.4)	55	SAD	STAI-T:46.45	DSM-IV	17	25.7 (7.2)	58.8	STAI-T:26	SPM5	3 T	8	AlphaSim	AAL atlas	10.5
7	Jung et al., 2018	36	25.4 (3.1)	52.8	SAD	LSAS:78.3	DSM–5	42	24.7 (3.1)	54.8	LSAS:18	AFNI	3 T	4	FWE	FreeSurfer N27	9.5
8	Ergül et al., 2019	34	28.2 (6.3)	70.6	SAD	LSAS:75.11	DSM-IV	21	27.2 (6.4)	61.9	NA	SPM8	3 T	8	FWE	AAL atlas	11.5
9	Du et al., 2021	38	41.1 (11.2)	73.6	GAD	SAI:53.89	DSM-IV	20	39.2 (8.2)	85.0	SAI:34.05	SPM8	3 T	NA	AlphaSim	SPM anatomy toolbox	9
10	Toazza et al., 2016	18	17.9 (2.5)	42.1	GAD, SAD and SepAD	NA	DSM-IV	19	16.7 (2.3)	50.0	NA	AFNI	3 T	6	AlphaSim	Probability mask	9.5
11	Wang et al., 2021	37	39.8 (9.4)	72.9	GAD	SAI:51.86	DSM-IV	31	36.2 (9.2)	77.4	SAI:31.55	SPM12	3 T	8	FWE	FreeSurfer N27	9.5
12	Arnold Anteraper et al., 2014	17	24.7 (6.3)	52.9	SAD	LSAS:77.9	DSM-IV	17	25.0 (7.5)	52.9	LSAS	SPM8	3 T	NA	FWE	5 mm sphere	8.5
13	Roy et al., 2013	15	14.9 (1.7)	66.7	GAD	SCARED:30.3	DSM-IV	20	14.8 (1.7)	65.0	SCARED:5.6	AFNI	3 T	NA	GRF	4 mm sphere	11
14	Mizzi et al., 2024	42	27.6 (7.5)	50.0	SAD	LSAS:81.2	DSM-IV	93	26.1 (6.5)	52.7	LSAS	SPM12	3 T	8	FWE	Probability mask	10

*Note:* Quality score was derived from a 12-point Imaging Methodology Quality Assessment Checklist (see Supplementary Material 2). ADs, anxiety disorders; HC, healthy controls; Age, mean age (SD), years; SD, standard deviation; GAD, generalized anxiety disorder; SAD, social anxiety disorder; SepAD, separation anxiety disorder; NA, not available; STAI, State–Trait Anxiety Inventory; PARS, Pediatric Anxiety Rating Scale; HAMA, Hamilton Anxiety Scale; SCARED, Screen for Child Anxiety-Related Emotional Disorders; PSWQ, Penn State Worry Questionnaire; FQ, Fear Questionnaire; STAI-T, State–Trait Anxiety Inventory–Trait; LSAS, Liebowitz Social Anxiety Scale; SAI, State Anxiety Inventory; DSM, Diagnostic and Statistical Manual of Mental Disorders; FWE, family-wise error; GRF, Gaussian random field; AAL, automated anatomical labeling.

### Meta-analysis

The voxel-based neuroimaging meta-analysis was conducted using SDM-PSI (Seed-based d Mapping with Permutation of Subject Images, version 6.23, http://www.sdmproject.com/software), a validated statistical technique that meta-analyzes peak coordinates to identify differences in brain activity or structure (Radua et al., 2012). Before analysis, all coordinates were converted from Talairach space to MNI space, and *p*-values were transformed into *t*-values.

The SDM-PSI procedure involved six steps. First, peak coordinates and corresponding *t*-values for significant amygdala-based whole-brain rsFC differences between patients and healthy controls were extracted for each study and recorded in separate files. Demographic data were compiled in an SDM table. To avoid a voxel being coded in opposite directions, positive and negative coordinates were reconstructed on a single map (Radua et al., 2012). Second, preprocessing was performed to generate the maps of the lower and upper bounds of possible effect sizes for each study separately based on the peak coordinate information using a gray matter mask, anisotropy of 1, isotropic full-width half maximum (FWHM) of 20 mm, and a voxel size of 2 mm. Third, a mean analysis was conducted using the MetaNSUE algorithms to estimate the map of the most likely effect size and its standard error (Albajes-Eizagirre, Solanes, & Radua, 2019; Radua et al., 2015), adding normal spatially correlated noise to the maximum likely effect sizes (Albajes-Eizagirre et al., 2019; Radua et al., 2012), and multiple imputations of the maps were created based on these estimations and the bounds. Fourth, the imputed effect size maps from each dataset were combined using a standard random-effects model, accounting for sample size, intra-study variability, and between-study heterogeneity (Radua et al., 2012). Rubin’s rules were applied to pool the multiple imputations (Albajes-Eizagirre et al., 2019). Fifth, the mean map was a family-wise error (FWE) corrected through permutation tests of the subject images, generating a distribution of the maximum statistic. Finally, threshold-free cluster enhancement (TFCE) was used to threshold the results of the main analysis (*P* < 0.005, extent threshold of 50 voxels) (Smith & Nichols, 2009). If no statistically significant group differences were identified, uncorrected results (*p* < 0.005, extent threshold of 50 voxels) were employed to explore possible trend-level group differences in rsFC. Full methodological details are described in prior publications and the SDM-PSI reference manual (https://www.sdmproject.com/manual/) (Albajes-Eizagirre et al., 2019).

### Subgroup analysis

To control age differences across studies, we performed an adult subgroup analysis that included only studies with a mean age > 18 years. The number of non-adult studies is too small to conduct a subgroup analysis. Consistent with the primary analyses, all results were thresholded at an uncorrected voxel-wise *p* < 0.005 with a cluster extent of at least 50 voxels. In addition, to examine potential hemispheric differences in amygdala connectivity, we conducted subgroup meta-analyses separately for studies using the left versus right amygdala as the seed region.

### Sensitivity analysis

Study heterogeneity and replicability were assessed using a whole-brain voxel-based jackknife sensitivity analysis with the leave-one-out method. The main analysis was repeated 15 times, each time excluding a different dataset. Brain regions that remained significant across all or most iterations were considered highly robust and replicable (Aoki, Cortese, & Tansella, 2015; Zhang, Picchioni, Allen, & Toulopoulou, 2016).

### Meta-regression

We performed meta-regression to explore the potential effects of clinical characteristics and demographics on the strength of linear and quadratic trends, including the mean age, the percentage of female patients, and symptom severity (percentage of anxiety scores to total scale scores on specific anxiety scales). This regression analysis used a threshold of *p* < 0.0005, clusters >50 voxels. It required that the brain regions identified be among those found in the meta-analysis results to minimize the false positive rate to the greatest extent possible.

### Heterogeneity analysis and publication bias

We extracted peak coordinates from included studies to assess heterogeneity and potential publication bias. Heterogeneity of significant voxels was evaluated using the *I* ^2^ statistic, with values <50% indicating low heterogeneity. Publication bias was examined using funnel plots and Egger’s test within SDM, with *p* < 0.05 indicating significant bias (Egger, Smith, & Phillips, 1997).

## Results

### Included studies and sample characteristics

Our search identified 14 amygdala-based rsFC studies comprising 15 independent datasets (11 in adults and 4 in adolescents) that met the predefined inclusion and exclusion criteria. The pooled sample consisted of 378 individuals with anxiety disorders and 405 HCs. Detailed demographic and clinical characteristics are summarized in Table 1, and scanning parameters with corresponding analytic methods are provided in Supplementary Table S1.

### Abnormal seed (amygdala)-based rsFC in anxiety disorder patients (vs. HC)

**Figure 2.**
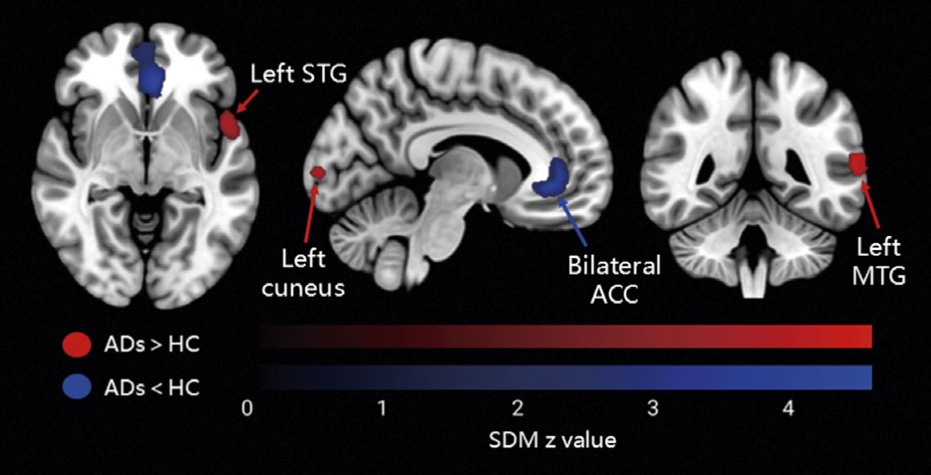
Meta-analysis results of abnormal resting-state functional connectivity (rsFC) with seeds in the amygdala (*P* < 0.005, extent threshold of 50 voxels). *Note:* ADs, anxiety disorders; HC, healthy control; MTG, middle temporal gyrus; STG, superior temporal gyrus; ACC, anterior cingulate cortex.

**Figure 3.**
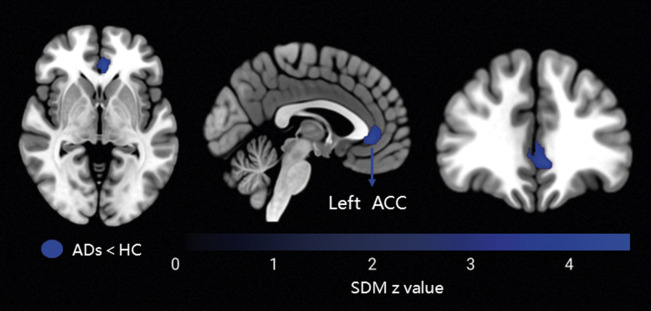
Meta-analysis results of TFCE-corrected abnormal resting-state functional connectivity (rsFC) with seeds in the amygdala (*P* < 0.005, extent threshold of 50 voxels). *Note:* ADs, anxiety disorders; HC, healthy control; ACC, anterior cingulate cortex; TFCE, threshold-free cluster enhancement.

Relative to HCs, patients with anxiety disorders showed decreased functional connectivity between the amygdala and the bilateral ACC (*g* = −0.54, 95% confidence interval [CI]: −0.73 to −0.35), extending into portions of the SFG. In contrast, increased connectivity was observed between the amygdala and the left STG (*g* = 0.46, 95% CI: 0.27–0.65), including parts of the insula, as well as the left MTG (*g* = 0.38, 95% CI: 0.19–0.57) and left cuneus (*g* = 0.35, 95% CI: 0.17–0.53; Figures 2 and 3, and Table 2). After TFCE correction, only reduced amygdala – left ACC (*g* = −0.54, 95% CI: −0.73 to −0.35) connectivity remained statistically significant (corrected *p* = 0.001; Figure 3 and Table 2).

**Table 2.** Meta-analysis results regarding regional differences in amygdala-based rsFC

Local maximum				Cluster		Jackknife sensitivity analysis, Survival rate	Egger test,*P*-value
Region	MNI coordinates	SDM-Z	*p*-value	No. of voxels	Breakdown (No. of voxel)		
**ADs vs. HC, TFCE-corrected (No. of datasets: 15)**							
Decreased connectivity(ADs < HC)							
Left ACC	−6, 34, −4	−5.555	0.002	160	Left ACC (142); Right ACC (18);	12 out of 15	0.287
**ADs vs. HC, uncorrected (No. of datasets: 15)**							
Increased connectivity (ADs > HC)							
Left STG	−52, 10, −8	4.776	<0.000001	259	Left STG (234); Left insula (25);	15 out of 15	0.849
Left MTG	−60, −46, 12	3.880	0.000052	167	Left MTG (101); Left STG (66);	13 out of 15	0.596
Left cuneus	−8, −96, 4	3.924	0.000044	53	Left cuneus (38); Left superior occipital gyrus (15);	13 out of 15	0.611
Decreased connectivity (ADs < HC)							
Bilateral ACC	−6, 34, −4	−5.555	<0.000001	816	Left ACC (378); Right ACC (109); Right SFG (184); Left SFG (145);	15 out of 15	0.287

*Note:* rsFC, resting-state functional connectivity; MNI, Montreal Neurological Institute; SDM, seed-based d mapping; ADs, anxiety disorders; HC, healthy control; ACC, anterior cingulate cortex; STG, superior temporal gyrus; MTG, middle temporal gyrus; SFG, superior frontal gyrus.

### Subgroup analysis

Compared with HCs, adults with anxiety disorders (*k* = 11 datasets) demonstrated amygdala functional connectivity patterns largely consistent with the main analysis (*k* = 15 datasets, including 4 adolescent samples). Specifically, there was increased functional connectivity between the amygdala and the left MTG and STG. There was also decreased connectivity between the bilateral ACC and the bilateral SFG. After TFCE correction, only the reduced connectivity between the amygdala and left ACC was significant (Supplementary Table S2 and Supplementary Figure S1).

Subgroup analysis revealed distinct FC patterns of the left and right amygdala in anxiety disorders (*k* = 14 datasets, with 1 dataset not using the left and right amygdala as separate seed regions). Compared to HC, the left amygdala showed increased connectivity with the left cuneus and decreased connectivity with the bilateral ACC, including the bilateral SFG. After TFCE correction, only the reduced connectivity between the left amygdala and left ACC was significant (Supplementary Table S2 and Supplementary Figure S2). The right amygdala showed increased connectivity with the left STG, with no significant areas after TFCE correction (Supplementary Table S2 and Supplementary Figure S3).

### Sensitivity test for the main findings

A jackknife analysis was conducted to examine the heterogeneity of the included studies. Whole-brain jackknife sensitivity analysis showed that rsFC between the amygdala and left ACC was highly replicable, with 12 combinations of studies preserved after TFCE correction. In the case of uncorrected analysis, the ACC and left STG results remained significant across all 15 dataset combinations. Connectivity between the left cuneus and the extension from the left MTG to the left STG was significant in all but two combinations (Supplementary Table S3). These findings remained significant across all or the vast majority of study combinations, indicating that our results are not driven by any single study.

### Meta-regression analyses

Meta-regression analysis did not find a significant correlation between the amygdala FC abnormalities and age, gender (percentage of females), or severity of anxiety in patients with anxiety disorders.

### Heterogeneity analysis and publication bias

The heterogeneity analysis showed a low *I* ^2^ index (2.36–9.42%), indicating no significant heterogeneity across studies. Egger’s tests were nonsignificant for all identified clusters (*p* > 0.05), denoting the absence of asymmetry in the funnel plots (no larger effect size in small studies), suggesting that there is no significant publication bias in the study results (Supplementary Table S4 and Supplementary Figure S4) (Ranzini et al., 2022).

## Discussion

**Figure 4.**
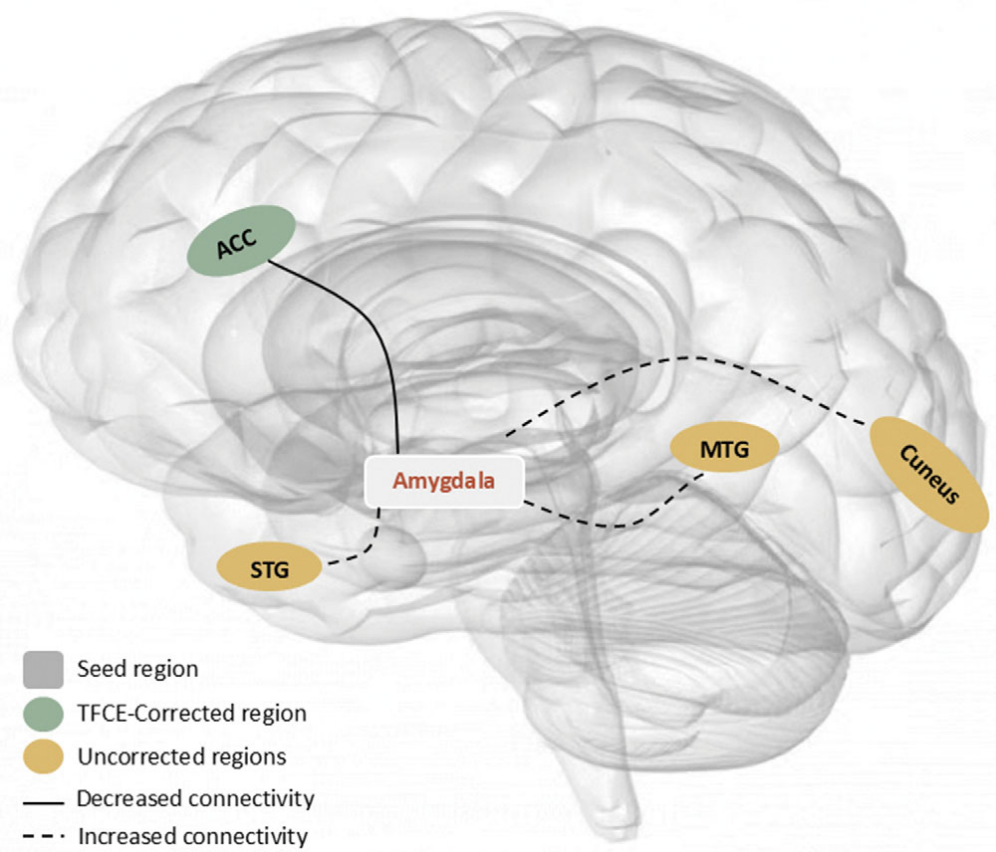
Patterns of amygdala-based functional connectivity in patients with anxiety disorders. *Note:* MTG, middle temporal gyrus; STG, superior temporal gyrus; ACC, anterior cingulate cortex.

Disrupted communication between the brain’s emotion hub, the amygdala, and its regulatory centers may be a defining neural signature of anxiety disorders. Using SDM-PSI, we synthesized whole-brain amygdala FC data across studies and found that, compared with healthy controls, patients with anxiety disorders showed markedly reduced resting-state FC between the amygdala and the ACC, in addition to increased connectivity with the left superior and middle temporal gyri and the cuneus. Importantly, only the amygdala–ACC reduction – driven predominantly by the left amygdala – remained significant after rigorous TFCE correction, highlighting its potential as a robust biomarker. Subgroup analyses in adults yielded consistent results, underscoring the reproducibility and specificity of this circuit abnormality. Jackknife sensitivity and heterogeneity assessments further confirmed the stability of these findings. This work not only refines the neurofunctional connectivity model of anxiety disorders but also points toward a plausible neural target for future biomarker development and intervention strategies (Figure 4).

In this meta-analysis, reduced FC between the amygdala and the left ACC emerged as the most statistically significant finding. A diffusion tensor imaging study in individuals with GAD revealed reduced fractional anisotropy in the bilateral uncinate fasciculus, the primary structural connection between ACC and amygdala, raising the possibility of compromised structural connectivity between these two regions, which may in turn accentuate the observed reductions in functional connectivity between them (Tromp et al., 2012). This is further supported by a tract-tracing study in rhesus monkeys, which demonstrated that subregions of the ACC project densely and in a laminar-specific manner to the amygdala (Ghashghaei, Hilgetag, & Barbas, 2007). The observed decreased FC between the amygdala and ACC in individuals with anxiety disorders aligns with the possibility of a top-down emotion dysregulation pathway, which posits that weakened prefrontal control leads to insufficient inhibition of amygdala reactivity, resulting in emotional dysregulation (Mochcovitch, da Rocha Freire, Garcia, & Nardi, 2014; Roy et al., 2009). A conjunction analysis in patients with anxiety disorders revealed that the FC between amygdala and ACC was reduced across both resting-state and threat-related conditions, reflecting that disrupted amygdala-ACC connectivity is a stable trait but not a variable state of anxiety disorders. From a large-scale brain network perspective, the ACC identified in our meta-analysis is a core component of the default mode network (DMN). Accordingly, our study suggests significantly decreased amygdala-DMN connectivity in anxiety disorders, consistent with prior reviews reporting decreased amygdala–DMN connectivity in association with increased anxiety in major depressive disorder (Briley et al., 2022). In summary, our study suggested that the disrupted amygdala–ACC FC was the most robust finding in anxiety disorders, which may be associated with impaired top-down regulation by the ACC over the amygdala, contributing to the uncontrollable fear and worry characteristic of anxiety disorders. Moreover, this disrupted pathway may serve as a translational target, consistent with emerging mechanism-based interventions for anxiety and depression that increasingly aim to modulate dysfunctional neurocircuitry (Sylvester, Luby, & Pine, 2024).

Importantly, these findings also align with treatment studies showing that effective interventions can normalize amygdala-prefrontal connectivity in anxiety disorders. For instance, CBT and SSRIs in youth increase rostral ACC activation during emotional conflict tasks, with greater activation predicting clinical improvement (Burkhouse et al., 2018). Reduced baseline activation in the dorsal ACC and dorsomedial PFC has also been shown to predict greater treatment response, suggesting that prefrontal deficits may represent a modifiable vulnerability (Burkhouse et al., 2017). Consistent with this, double-blind, placebo-controlled trials in adolescents demonstrated that escitalopram rapidly enhanced amygdala–ventrolateral and amygdala–vmPFC connectivity, with early changes strongly predicting subsequent symptom improvement (Lu et al., 2021, 2022). Together, these results suggest that disrupted amygdala–ACC and amygdala–PFC pathways are not only stable markers of anxiety disorders but also treatment targets, highlighting their relevance for both mechanism-based interventions and biomarker development.

We also observed increased connectivity between the amygdala and the STG, extending to the insula and MTG in individuals with anxiety disorders, although these findings did not survive the TFCE correction. The STG is involved in language comprehension and emotional perception, and cognitive aspects of fear processing (Quirk, Armony, & LeDoux, 1997; Phillips et al., 1998). One study suggested that patients with anxiety disorders may be more sensitive to scanner noise during resting-state MRI, leading to increased amygdala-STG co-activation as the brain processes this aversive stimulus (Liu et al., 2015). Such increased rsFC could contribute to heightened reactivity to sensory input and a negatively biased interpretation of information. The MTG subserves audiovisual integration and visual perception (Li et al., 2013; Li et al., 2016). It shares anatomical proximity and functional integration with the STG. Increased FC between the amygdala and MTG may reflect an enhanced susceptibility to inaccurate interpretation of stimuli (Pannekoek et al., 2013).

We also found increased amygdala-insula connectivity. The insula subserves interoceptive awareness and emotional processing (Phan, Wager, Taylor, & Liberzon, 2002), integrating bodily discomfort signals and transmitting them to the amygdala (Baur, Hanggi, Langer, & Jancke, 2013). Increased connectivity in this circuit may contribute to the core phenomenology of anxiety – specifically, amplified perception of bodily sensations (e.g. palpitations and shortness of breath) and their rapid appraisal as threatening. This pattern is consistent with findings in adolescents with GAD showing increased amygdala-insula connectivity during emotional face processing (Strawn et al., 2012). Further, an fMRI study in adults with GAD showed co-activation and increased amygdala and insula activity during emotional processing tasks (Buff et al., 2016). Taken together, the increased connectivity between the amygdala and the STG, MTG, and insula may represent a neural substrate for heightened vigilance to external cues, exaggerated emotional reactivity, and a negatively biased interpretation of sensory and interoceptive information – processes that maintain and intensify anxiety symptoms in clinical populations (Domschke, Stevens, Pfleiderer, & Gerlach, 2010).

Our findings demonstrate FC lateralization of the amygdala in anxiety disorders; that is, hemispheric differences in the co-activation patterns between the amygdala and other brain regions (Ocklenburg, Peterburs, & Mundorf, 2022). Across both corrected and uncorrected analyses, abnormal rsFC was predominantly observed in the left hemisphere, particularly between the left amygdala and left ACC. Subgroup analyses confirmed this pattern: decreased rsFC between the left amygdala and the left ACC, whereas the right amygdala analyses yielded no statistically significant results. This suggests that amygdala dysconnectivity in anxiety disorders is largely driven by the left amygdala, with left amygdala–left ACC hypoconnectivity potentially representing a neuroimaging marker of these conditions. Previous task-based meta-analyses extensively showed amygdala activity lateralization, consistently demonstrating that the left amygdala is more frequently activated than the right amygdala during emotional processing tasks (Baas et al., 2004; Sergerie et al., 2008). Evidence indicates that top-down regulatory processes may predominantly involve the left amygdala (Ochsner et al., 2009). The neurobiological mechanism underlying the lateralization of amygdala activity and connectivity needs to be explored by future studies.

A key strength of our study is the use of the updated SDM-PSI meta-analytic approach, which allowed us to incorporate both significant and nonsignificant findings, thereby reducing selection bias and increasing the robustness of our conclusions. Nevertheless, several limitations should be acknowledged. First, our analyses focused on rsFC changes of the amygdala solely – the most interested and widely invested brain region in anxiety disorders, but no other brain regions, like bed nucleus of the stria terminalis (BNST), which is closely associated with certainty and threat anticipation (Buff et al., 2017; Yassa, Hazlett, Stark, & Hoehn-Saric, 2012). Future studies incorporating more anxiety-associated regions may further refine the core pathology of anxiety disorders. Second, the current study did not investigate parallel neurostructural changes or task-based FC alterations. Integrating structural imaging studies, such as structural MRI and diffusion tensor imaging, could elucidate the structural alterations underlying FC changes. A multimodal imaging meta-analysis may offer a more comprehensive understanding of the neuropathoetiology of anxiety disorders. Third, the current meta-analysis cannot exclude potential bias that may result from different amygdala seed definition strategies (e.g. some used a coordinate of the amygdala to generate a sphere, some used a part or subregion of the amygdala, some used the whole left or right amygdala, etc.). Further studies should systematically subdivide the amygdala to clarify which specific subregions contribute to the abnormal coupling of amygdala–ACC, and better understand the roles of different amygdala subregions in anxiety disorders. Fourth, due to the limited number of studies and insufficient reporting of key clinical information (e.g. medication type and comorbidities), we were unable to explore the effects of medication and comorbidities or to compare FC differences between adolescents and adults with anxiety disorders or between GAD and SAD.

## Conclusion

In this meta-analysis, we applied advanced analytic methods (SDM-PSI) to characterize abnormal amygdala rsFC in anxiety disorders. We identified a robust reduction in rsFC between the left amygdala and the left ACC, underscoring the central role of the amygdala–ACC pathway in the neurobiology of anxiety. Subgroup analyses further identified lateralization of FC abnormalities, indicating that disruptions primarily involved the left hemisphere. Given the ACC’s role in top-down regulation of threat perception and emotional reactivity (Etkin, Egner, & Kalisch, 2011; Goldin et al., 2009), these findings link a specific neural circuit to core phenomenological features of anxiety – such as heightened threat vigilance, impaired fear extinction, and difficulty modulating distress. This meta-analysis refines our mechanistic understanding of anxiety disorders and identifies the amygdala–ACC pathway as a potential biomarker and therapeutic target for future, circuit-focused interventions.

## Supporting information

10.1017/S0033291726104310.sm001 Lu et al. supplementary material Lu et al. supplementary material

## Supplementary material

The supplementary material for this article can be found at http://doi.org/10.1017/S0033291726104310.
