# Supplementary material for: Altered amygdala resting-state functional connectivity in anxiety disorders: a coordinate-based meta-analysis
Source: Psychol Med. 2026 May 21;56:e161. doi: 10.1017/S0033291726104310 (PMC13200151; doi:10.1017/S0033291726104310)
Supplement: Lu et al. supplementary material [file S0033291726104310sup001.docx]

**Abnormal resting-state functional connectivity of amygdala in patients with anxiety disorders: A** **coordinate-based imaging meta-analysis**

**Supplemental material**

**Supplement 1. Literature Search Strategy**

We searched for studies that investigated alterations in resting-state functional connectivity (rsFC) of the amygdala in patients with anxiety disorders. According to the pre-registered protocol (https://www.crd.york.ac.uk/PROSPERO/view/CRD420251019885), we conducted the literature search in PubMed (<https://pubmed.ncbi.nlm.nih.gov/>), Web of Science (<https://apps.webofknowledge.com>), and Embase (<https://gateway.ovid.com>). The search strategy combined MeSH terms and keywords related to anxiety disorders and amygdala-based functional connectivity. The search terms included: (anxiety OR anxious) AND (rest*) AND (connect*) AND (amygdala*). To ensure completeness and consistency with prior literature, we also manually screened the reference lists of eligible studies and relevant meta-analyses to identify additional records.

**Supplement 2. Quality Assessment Checklist**

We used the Imaging Methodology Quality Assessment Checklist to assess the quality of included articles objectively and to identify potential sources of bias, thereby facilitating appropriate interpretation of the meta-analytic findings (Shepherd, Matheson, Laurens, Carr, & Green, 2012). The 12-point checklist is modified from Shepherd et al. with three categories: subject characteristics (items 1-4), methods for image acquisition and analysis (items 5-10), and results and conclusions (items 11-12). The items on the checklist were revised to meet the purposes of our meta-analysis and to enhance the transparency of reports. The items are as follows:

Category 1: Subject characteristics

1. Patients with anxiety disorders were evaluated prospectively, specific diagnostic criteria were applied, and demographic data were reported.

2. Healthy control subjects were evaluated prospectively, psychiatric and medical illnesses were excluded, and demographic data were reported.

3. Important variables or details (e.g., diagnostic criteria, medication status, past illness history) were examined either by stratification or statistical control.

4. Sample size per group > 10, and no significant difference in age and sex existed.

Category 2: Methods for image acquisition and analysis

5. Magnet strength at least 1.5T, and imaging acquisition parameters were clearly reported and appropriate for rs-fMRI analyses.

6. Whole-brain functional connectivity analysis was conducted without a priori regional selection beyond the predefined seed.

7. Peak coordinates were reported in a standard space.

8. The imaging technique used was clearly described so as it could be reproduced.

9. Measurements were clearly described so that they could be reproduced.

10. Results were corrected for multiple comparisons.

Category 3: Results and conclusions

11. Statistical parameters for significant and important non-significant differences were provided.

12. Conclusions were consistent with the results obtained and the limitations were discussed.

Note: Each item received a score of 1, 0.5 or 0 according to the criteria that were fully, partially or not met, respectively. The assessment score for each included study was reported in Table 1.

**Table S1.** Summary of methodological information of studies included in the meta-analysis

| **Study** | **Scanner** | **Scan Duration** | **TR/TE**  **(ms)** | **EO/C** | **Software** | **Smoothing**  **(FWHM)** | **Coordinate**  **System** |
| --- | --- | --- | --- | --- | --- | --- | --- |
| Hamm et al.2014 | 3T | 8 min | 2000/25 | Open | SPM8 | 8 mm | MNI |
| Li et al.2016(open) | 3T | 8 min | 2000/25 | Open | SPM8 | 9.5 mm | MNI |
| Li et al.2016(closed) | 3T | 8 min | 2000/25 | Closed | SPM8 | 9.5 mm | MNI |
| Liu et al.2015 | 3T | NA | 2000/20 | Closed | DPARSF2.0 | NA | MNI |
| Makovac et al.2015 | 1.5T | 5 min | 2520/43 | Open | SPM8 | NA | MNI |
| Pannekoek et al.2012 | 3T | 7.51 min | 2300/30 | Closed | FSL | 6 mm | MNI |
| Prater et al.2012 | 3T | 5 min | 2000/25 | Open | SPM5 | 8 mm | MNI |
| Jung et al.2018 | 3T | 5 min | 2000/30 | Open | AFNI | 4 mm | Talairach |
| Ergül et al.2019 | 3T | 7 min, 31 s | 2000/30 | Closed | SPM8 | 8 mm | MNI |
| Yang et al.2021 | 3T | NA | 2000/30 | Closed | SPM8 | NA | MNI |
| Toazza et al.2016 | 3T | 7 min, 46 s | 2000/ 30 | Open | AFNI | 8 mm | MNI |
| Wang et al. 2021 | 3T | NA | 2000/30 | Closed | SPM12 | 8 mm | MNI |
| Anteraper et al.2013 | 3T | 6 min, 24 s | 6000/30 | Open | SPM8 | NA | Talairach |
| Roy et al.2012 | 3T | 6 min | 2000/30 | Open | AFNI | NA | MNI |
| Mizzi et al.2024 | 3T | 8min, 38s | 1020/30 | Open | SPM12 | 8 mm | MNI |

**Abbreviations:** TR, repetition time; TE, echo time; EO, eyes open; EC, eyes close; FWHM, full-width at half-maximum; FSL, FMRIB Software Library; MNI, Montreal Neurological Institute; SPM, Statistical Parametric Mapping; DPARFS, Data Processing Assistant for Resting-State fMRI; AFNI, Analysis of Functional Neuroimages; NA, none.

**Table S2.** Subgroup meta-analysis results regarding regional differences in amygdala-based rsFC

| **Local maximum** | | | | |  | **Cluster** | |
| --- | --- | --- | --- | --- | --- | --- | --- |
| **Region** | **MNI coordinates** | | **SDM-Z** | **p-value** |  | **No. of voxels** | **Breakdown (No. of voxel)** |
| **Samples from ADs adults (No. of datasets:11)** | | | | | | | |
| **ADs vs. HC, TFCE-corrected** | | | | | | | |
| ***Decreased connectivity (ADs＜HC)*** | | | | | | | |
| Left ACC | | -4,36,-4 | -5.624 | 0.000999 |  | 51 | Left ACC(51); |
| **ADs vs. HC, uncorrected** | | | | | | | |
| ***Increased connectivity (ADs＞HC)*** | | | | | | | |
| Left MTG | | -64,-50,12 | 3.748 | 0.000089 |  | 147 | Left MTG(84);Left STG(63); |
| Left STG | | -50,6,-6 | 3.358 | 0.000392 |  | 76 | Left STG(76); |
| ***Decreased connectivity (ADs＜HC)*** | | | | | | | |
| Bilateral ACC | | -4,36,-4 | -5.624 | ＜0.000001 |  | 492 | Left ACC(330);Right ACC(101);Left SFG(29);Right SFG(26); |
| **Samples from left amygdala in ADs patients (No. of datasets:14)** | | | | | | | |
| **ADs vs. HC, TFCE-corrected** | | | | | | | |
| ***Decreased connectivity (ADs＜HC)*** | | | | | | | |
| Left ACC | | -6,34,-4 | -5.790 | 0.000999 |  | 115 | Left ACC(115); |
| **ADs vs. HC, uncorrected** | | | | | | | |
| ***Increased connectivity (ADs＞HC)*** | | | | | | | |
| Left cuneus | | -8,-96,4 | 3.772 | 0.000081 |  | 18 | Left cuneus(18); |
| ***Decreased connectivity (ADs＜HC)*** | | | | | | | |
| Left ACC | | -6,34,-4 | -5.790 | ＜0.000001 |  | 944 | Left ACC(395);Right SFG(259);Left SFG(180);Right ACC(110); |
| **Samples from right amygdala in ADs patients (No. of datasets:14)** | | | | | | | |
| **ADs vs. HC, uncorrected** | | | | | | | |
| ***Increased connectivity (ADs＞HC)*** | | | | | | | |
| Left STG | | -54,12,-10 | 3.579 | 0.000172 |  | 69 | Left STG(69); |

**Abbreviations:** rsFC, resting-state functional connectivity; MNI, Montreal Neurological Institute; SDM, seed-based d mapping; ADs, anxiety disorder; HC, healthy control; ACC, anterior cingulate cortex; STG, superior temporal gyrus ; MTG, middle temporal gyrus; SFG, superior frontal gyrus.

**Table S3.** Results of the jackknife sensitivity analysis of samples (No. of datasets:15, p=0.005, cluster size >50 voxels).

| **Discarded analysis** | **Uncorrected** | | | | **TFCE-corrected** |
| --- | --- | --- | --- | --- | --- |
|  | Left superior temporal gyrus (259) | Left middle temporal gyrus (167) | Left cuneus (53) | Left anterior cingulate cortex (816) | Left anterior cingulate cortex (160) |
| Hamm et al. | Yes | Yes | No | Yes | Yes |
| Li et al. eyes-open. | Yes | Yes | Yes | Yes | Yes |
| Li et al. eyes-closed. | Yes | Yes | Yes | Yes | Yes |
| Liu et al. | Yes | Yes | Yes | Yes | Yes |
| Makovac et al. | Yes | Yes | Yes | Yes | Yes |
| Pannekoek et al. | Yes | No | Yes | Yes | Yes |
| Prater et al. | Yes | Yes | Yes | Yes | No |
| Jung et al. | Yes | Yes | Yes | Yes | Yes |
| Ergül et al. | Yes | Yes | No | Yes | Yes |
| Yang et al. | Yes | No | Yes | Yes | No |
| Toazza et al. | Yes | Yes | Yes | Yes | Yes |
| Wang et al. | Yes | Yes | Yes | Yes | No |
| Anteraper et al. | Yes | Yes | Yes | Yes | Yes |
| Roy et al. | Yes | Yes | Yes | Yes | Yes |
| Mizzi et al. | Yes | Yes | Yes | Yes | Yes |
| **Survival Rate** | 15/15 | 13/15 | 13/15 | 15/15 | 12/15 |

**Table S4.** Results of the meta-analysis on amygdala resting-state functional connectivity (rsFC).

| **Cluster** | **MNI**  **(x,y,z)** | **SDM-Z** | **Hedges’g** | **I²** | **Egger**  **test p** | **AAL regions** | **Correction status** |
| --- | --- | --- | --- | --- | --- | --- | --- |
| **1** | -52,10,-8 | 4.776 | 0.46 | 3.80 | 0.849 | Left superior temporal gyrus | Uncorrected |
| **2** | -60,-46,12 | 3.880 | 0.38 | 9.42 | 0.596 | Left middle temporal gyrus | Uncorrected |
| **3** | -8,-96,4 | 3.924 | 0.35 | 2.36 | 0.611 | Left cuneus | Uncorrected |
| **4** | -6,34,-4 | -5.555 | -0.54 | 3.70 | 0.287 | Bilateral anterior cingulate cortex | Uncorrected |
| **5** | -6,34,-4 | -5.555 | -0.54 | 3.70 | 0.287 | Left anterior cingulate cortex | Corrected |

**Notes:** Cluster 5 represents the TFCE-corrected result corresponding to the same anatomical cluster identified under the uncorrected threshold (Cluster 4); therefore, effect size estimates are identical. MNI, Montreal Neurological Institute; SDM-Z, z score reported by the Seed-based d Mapping software; AAL, Automated Anatomical Labeling; Hedges’ g, effect size estimate; I², heterogeneity statistic; Egger test p, publication bias test.

The impact of the heterogeneity of the studies on results (I²) is low (ranging from 2.36% to 9.42%). The Egger’s test p for all 5 clusters is not significant, denoting the absence of an asymmetry in the funnel plots (i.e. no larger effect size in small studies), ruling out the possibility that small studies are only published if they find large effect sizes.

At the uncorrected threshold prior to FWE correction, the resulting meta-analytical map includes 5 clusters (size: from 53 to 816 voxels, z scores from -5.555 to 4.776). The main peaks are located in the left superior temporal gyrus (clusters 1, BA38), and left anterior cingulate cortex (cluster 4, BA11). Cluster 1, From the left superior temporal gyrus (MNI coordinates: -52,10, -8, BA38), extends to the insula (BA48). Cluster 2, whose main significant peak of activation is located within the left middle temporal gyrus (MNI coordinates: -60, -46,12, BA22), extends to the left superior temporal gyrus (BA22). Cluster 3, whose main significant peak activation is located within the left cuneus (MNI coordinates: -8, -96,4 BA17), extending to the left superior occipital gyrus (BA17). Cluster 4, whose main significant peak of reducing is located within the left anterior cingulate cortex (MNI coordinates: -6,34, -4 BA11), extends to the right anterior cingulate cortex (BA25) and superior frontal gyrus (BA10).

Specially, we found significantly decreased results in this brain region of cluster 5 after tfce-corrected. The size of this region is 144 voxels. Cluster 5, whose main significant peak of reducing is located within the Left anterior cingulate cortex (MNI coordinates: -6,34,-4 BA11), extends to the superior frontal gyrus (BA10).

**Figure S1.** Adult subgroup meta-analysis results of abnormal resting-state functional connectivity (rsFC) with seeds in the amygdala.

**Abbreviations:** ADs, anxiety disorder; HC, healthy control; MTG, middle temporal gyrus; STG, superior temporal gyrus; ACC, anterior cingulate cortex.


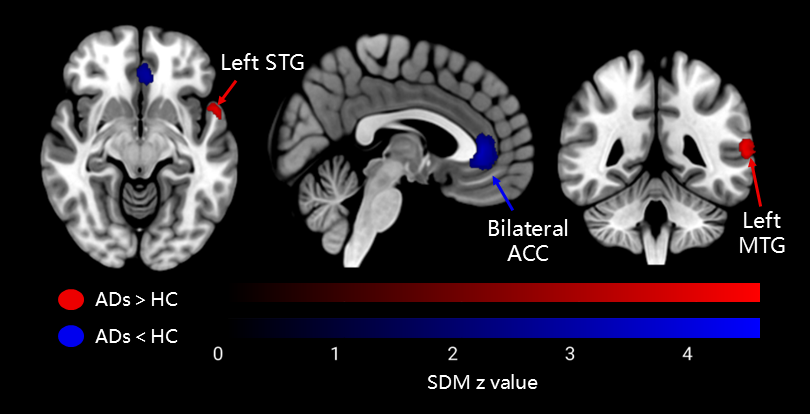


**Figure S2.** Subgroup meta-analysis results of abnormal resting-state functional connectivity (rsFC) with seeds in the left amygdala.

**Abbreviations:** ADs, anxiety disorder; HC, healthy control; ACC, anterior cingulate cortex.


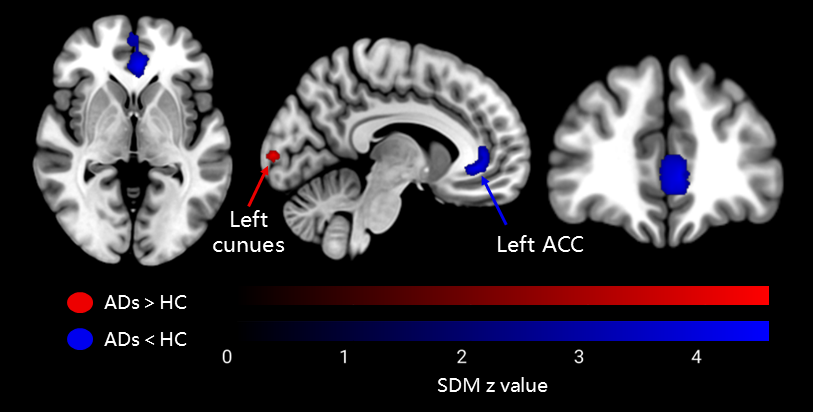


**Figure S3.** Subgroup meta-analysis results of abnormal resting-state functional connectivity (rsFC) with seeds in the right amygdala.

**Abbreviations:** ADs, anxiety disorder; HC, healthy control; STG, superior temporal gyrus.


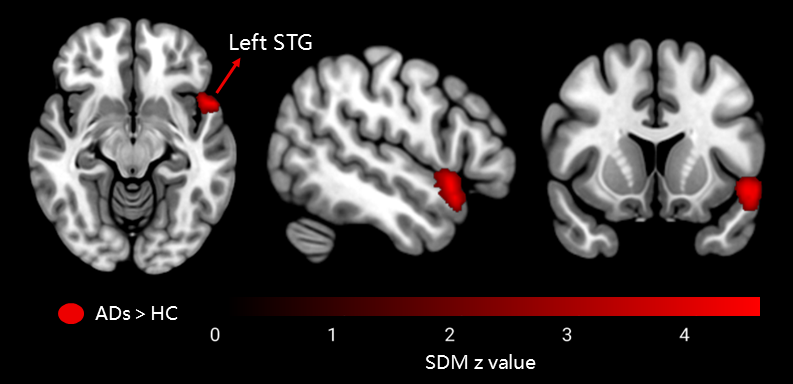


**Figure S4.** Results of funnel plot analysis to test for publication bias (seed in the amygdala).


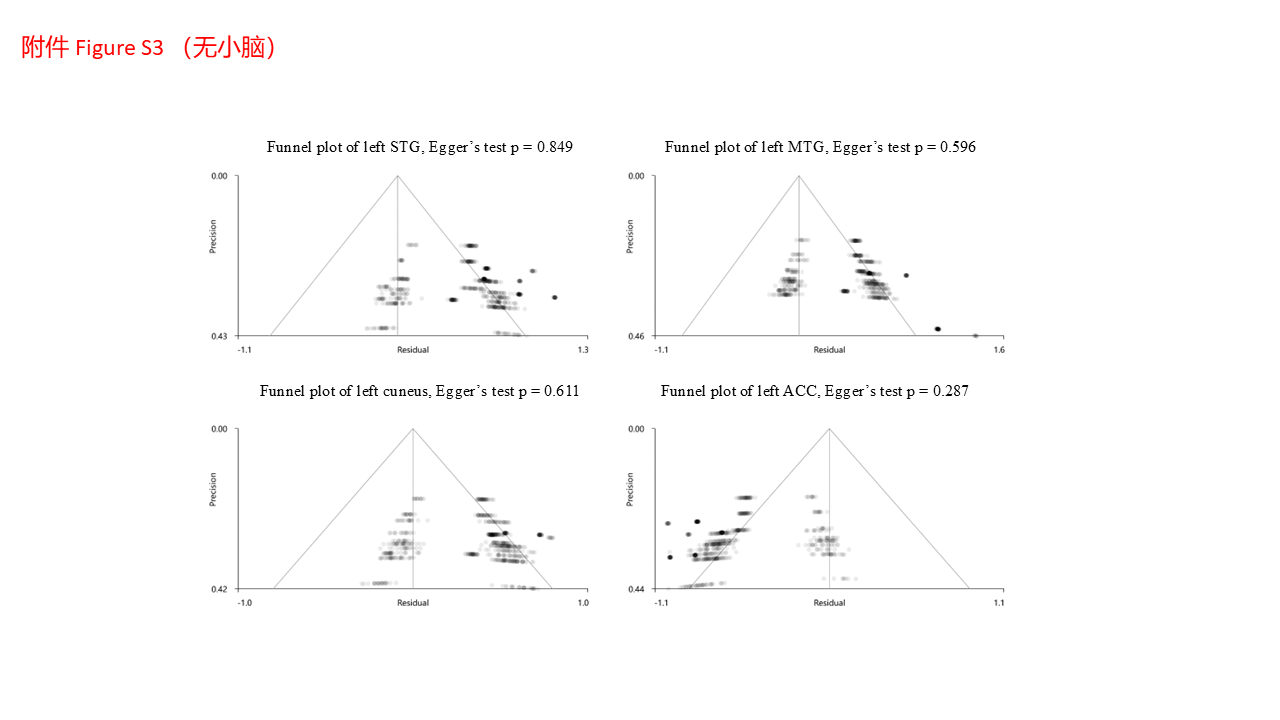


**Abbreviations:** STG, superior temporal gyrus; MTG, middle temporal gyrus; ACC, anterior cingulate cortex;

**Reference:**

Shepherd, A. M., Matheson, S. L., Laurens, K. R., Carr, V. J., & Green, M. J. (2012). Systematic meta-analysis of insula volume in schizophrenia. *Biol Psychiatry, 72*(9), 775–784. doi:10.1016/j.biopsych.2012.04.020
